# Predictors of compulsive cyberporn use: A machine learning analysis

**DOI:** 10.1016/j.abrep.2024.100542

**Published:** 2024-03-20

**Authors:** Farah Ben Brahim, Robert Courtois, Germano Vera Cruz, Yasser Khazaal

**Affiliations:** a University of Tours, QualiPsy, Tours, France; b Lausanne University, Lausanne, Switzerland; c Department of Psychology, UR 7273 CRP-CPO, University of Picardie Jules Verne, Amiens, France; d Addiction Medicine, Lausanne University Hospital; e Department of Psychiatry and Addictology, Montreal University, Canada

**Keywords:** Addiction, Cyberporn, Compulsive cyberporn use, Machine learning

## Abstract

**Introduction:**

Compulsive cyberporn use (CCU) has previously been reported among people who use cyberporn. However, most of the previous studies included convenience samples of students or samples of the general adult population. Research examining the factors that predict or are associated with CCU are still scarce.

In this study, we aimed to (a) assess compulsive cyberporn consumption in a broad sample of people who had used cyberporn and (b) determine, among a diverse range of predictor variables, which are most important in CCU scores, as assessed with the eight-item Compulsive Internet Use Scale adapted for cyberporn.

**Materials and Methods:**

Overall, 1584 adult English speakers (age: 18–75 years, M = 33.18; sex: 63.1 % male, 35.2 % female, 1.7 % nonbinary) who used cyberporn during the last 6 months responded to an online questionnaire that assessed sociodemographic, sexual, psychological, and psychosocial variables. Their responses were subjected to correlation analysis, analysis of variance, and machine learning analysis.

**Results:**

Among the participants, 21.96% (in the higher quartile) presented CCU symptoms in accordance with their CCU scores. The five most important predictors of CCU scores were related to the users’ strength of craving for pornography experiences, suppression of negative emotions porn use motive, frequency of cyberporn use over the past year, acceptance of rape myths, and anxious attachment style.

**Conclusions:**

From a large and diverse pool of variables, we determined the most important predictors of CCU scores. The findings contribute to a better understanding of problematic pornography use and could enrich compulsive cyberporn treatment and prevention.

## Highlights

• Limited research exists on factors predicting or related to CCU.
• According to the subjects' CCU scores, 21.96% showed signs of CCU.
• ML analysis identified the most important determinants of CCU scores.
• The most important predictor is the users’ strength of craving for pornography experiences.

## 1 Introduction

Since the early 2000 s, global cyberporn consumption has increased significantly (Lewczuk et al., 2022, Pornhub., 2022). It is the most common cybersex behavior (Fisher and Barak, 2001, Ross et al., 2012). Pornhub, one of the most popular pornographic websites, had 42 billion visits in 2019, an increase of 19 % from 2018. Using Polish “objective website traffic data”, Lewczuk et al. (2022) stated that between October 2004 and October 2016, the projected number of people accessing pornography online tripled. Mestre-Bach et al. (2020) reported that cyberporn consumption increased 61 %, 57 %, and 38 % in Spain, Italy, and France, respectively, during COVID-19.

People who engage in cyberporn may have a range of profiles. Adults, teens, men, and women may adopt this activity (De Alarcón et al., 2019, Efrati and Amichai-Hamburger, 2019, Emmers-Sommer, 2018, Levin et al., 2012). The mean age of Pornhub users in 2022 was 37 years, 18- to 24-year-olds being the most prevalent. The United States and the United Kingdom have the most Pornhub site users (Pornhub, 2019).

Although cyberporn is easier to use than other types of pornography, its use is still potentially maladjusted, as some studies reveal “addictive” or “compulsive” use (Allen et al., 2017, Chen and Jiang, 2020, De Alarcón et al., 2019), with increasing prioritization, loss of control, and negative impacts (Chen and Jiang, 2020, Cooper et al., 1999). Compulsive cyberporn use (CCU) continues to increase (Camilleri et al., 2021, Chen and Jiang, 2020, Griffiths, 2012, LeBlanc and Trottier, 2022, Wéry and Billieux, 2017). A Pornography-Masturbation-Orgasm (PMO) cycle seems to interfere with problematic cyberporn consumption (Burnett, 2022, Smith et al., 2022). Thus, some individuals who were considering CCU decided to join porn abstinence programs. They want to break the PMO loop and rediscover real sexuality over porn (Smith et al., 2022).

In recent years, CCU growth has been seriously investigated, mainly among students (e.g. Bulot et al., 2015, Döring et al., 2017, Kumar et al., 2021). Previous studies examined factors associated with problematic porn use, suggesting that, across populations, it is linked to male sex, young age, religion, frequent Internet use, negative emotions, and novelty seeking (Ballester-Arnal et al., 2017, Frangos et al., 2011, Ross et al., 2012; Štulhofer et al., 2016). Recent research has focused on psychopathological dimensions and a few CCU-related psychological features. Examples of factors that have been linked to CCU include depression, social anxiety, and impulsivity (Bőthe et al., 2019, Camilleri et al., 2021, Hakkim et al., 2022, Kumar et al., 2021, Studer et al., 2019).

The literature shows inconsistent conclusions about CCU predictors and related factors. Numerous studies have been conducted among people who are not specifically known to be cyberporn users. Other studies collected data on a variety of cybersex activities, but not specifically on cyberporn or compulsive use (e.g. Floyd & Grubbs, 2022). In addition, different variables are almost never compared within a single sample. More studies on a wide range of variables and a large sample of participants are needed to understand CCU. Even though, some studies advanced associations between compulsive cybersex and avoidant attachment (Efrati et al., 2021, Varfi et al., 2019), and between some media formats (especially violent pornography and general pornography) and rape myth acceptance (Hedrick, 2021). The importance of assessing this later association is suggested by the frequency of coercion related scenes among porn movies (Bridges et al., 2010, Carrotte et al., 2020) as well as by the reported association between sexual behaviors and trauma related sexual experiences. As far as we know, there is lack of information is available on the connection between CCU and attachment, arousal for certain porn styles, or coercive sexual relationship factors such as acceptance of the rape myth.

***The present study objective***.

In this study, we aimed to (a) assess CCU in a broad sample of people who had used cyberporn and (b) determine, among a diverse range of predictor variables, which are most important in CCU scores.

Two research questions (RQs) were addressed. RQ1: What is the distribution of CCU scores among people with cyberporn use? RQ2: What are the most important sociodemographic, sexual, psychological, and psychosocial variables that determine CCU scores?

As we used an exploratory study design, no hypotheses were made that were associated with the RQs.

## 2 Materials and methods

### 2.1 Participants and procedure

The study included 1584 online questionnaire respondents; of these, 26 % were American and 45.6 % British. Appendix 1 shows the details of the participants’ nationalities. An anonymous SphinxOnline survey was used. We searched for adults over 18 years who had watched pornography at least once in the past 6 months. They were recruited through Prolific (https://www.prolific.ac), an academic crowdsourcing service that provides high-quality data (Palan and Schitter, 2018, Peer et al., 2022). Meeting our selection criteria was possible since Prolific offered to pick pornography consumption over the past six months from its categories of hobbies. The study was advertised for using this ad “This study concerns the porn use by adult people. Its aim is to better understand the links with sexual attitudes and representations, sexual motivations, sexual desire, previous experiences (problematic or not), relations with his/her partner(s), etc. There are no right or wrong answers; Only your own answer counts. The procedures were approved by a university ethics committee”. The [anonymized] Research Ethics Committee (2020–04-05) approved the study, and all participants gave informed consent online. Recruitment occurred in May 2021.

### 2.2 Materials

Study variables included five predictor categories and one outcome variable. We evaluated 56 predictors related to each measure's dimensions and subscales.

#### 2.2.1 Outcome variable

CCU was measured with the eight-item short form of the Compulsive Internet Use Scale (CIUS) (Gmel et al., 2019; Meerkerk et al., 2009). CCU scores were measured on a 5-point scale, with a higher score indicating greater CCU. Gmel et al. (2019) noted that this unidimensional short form had good internal consistency. In 2019, Varfi et al. adapted the CIUS for cybersex; in our study, the CIUS was adapted to cyberporn (Ben Brahim et al., 2023), with “Internet” indicating pornographic sites.

#### 2.2.2 Predictor variables

##### 2.2.2.1 Socio-demographic characteristics

The characteristics included were age, gender, sexual orientation, and marital status.

##### 2.2.2.2 Cyberporn use patterns

The cyberporn use patterns included were as follows: weekly cyberporn use duration (CUD) (range: 0 to 40 h), frequency of cyberporn use (FCU) over the past year (11-point scale from “Never” to “More than 7 times a week”), whether participants were paying specific items (6-point scale from “Never” to “Every day”) or a subscription (yes or no) for their cyberporn use, negative moral perception of pornography (7-point scale from “Strongly disagree” to “Strongly agree”) with the following specific item from Grubbs et al. (2019) “I believe that pornography use is morally wrong.”, whether participants’ romantic and sexual lives improved since starting cyberporn use (5-point scale from “Not at all” to “Definitely yes” for each separate item), the degree of arousal for 10 pornographic styles (domination, humiliation, submission, romantic love, soft porn, groups with many males, groups with many females, young people, older people, stories, and dialogues) (4-point scale from “Very arousing” to “Not arousing at all”), and cyberporn use variations since the COVID-19 period started (7-point scale from “significantly increased” to “significantly decreased”).

We measured pornographic craving experience with the Strength of Pornography Craving Experience (PCE-S) scale (Ben Brahim et al., 2023). This measure adapts the “strength” of the Craving Experience Questionnaire to porn consumption (May et al., 2014). It uses the intrusion theory and comprises three dimensions (imagery, intensity, and intrusion), 10 items, and an 11-point scale from “Not at all” to “Extremely.” Higher scores suggest stronger porn cravings.

The Pornography Use Motivations Scale (PUMS) was used to evaluate motives for porn use (Bőthe et al., 2021). This 24-item (7-point scale) measure contains eight dimensions (sexual pleasure, sexual curiosity, fantasy, boredom avoidance, lack of sexual satisfaction, emotional distraction or suppression, stress reduction, self-exploration). Each participant receives eight scores, one for each dimension of the scale. Higher scores indicate greater endorsement of the relevant motive.

##### 2.2.2.3 Sexual dimensions

We also investigated sexual motives by using the Sexual Function Scale (SFS) (Nelson, 1978). This seven-dimensional instrument asks respondents why they perform sexually and how essential each reason is. In line with authors of previous studies (Abbey et al., 2006, Browning et al., 2000, Fortier, 2018), we deployed the items assessing dominance (eight items) and submission (eight items) on a 4-point scale. Each participant received two dimension-based scores. Higher scores suggest endorsement of the sexual motive.

The Sexual Desire Inventory (SDI) (Mark et al., 2018, Spector et al., 1996) measures solitary and dyadic sexual desire with 14 items (a 7-point scale and an 8-point scale). Each subject received dyadic and solitary sexual desire scores. Both dimensions of sexual desire increase with higher scores.

The number of sexual partners and frequency of intercourse for participants in the last 30 days was also examined. Their past-year sexual satisfaction was rated on a 9-point scale. Higher scores indicate sexual satisfaction. We also assessed participants' sexual self-esteem on a 4-point scale.

##### 2.2.2.4 Psychosocial and psychological dimensions

The Experiences in Close Relationships – Short Form (ECR-S), a brief variant of the Experiences in Close Relationships – Revised questionnaire (Fraley et al., 2000), examined attachment type with 12 items and a 7-point scale for anxious and avoidant attachment. Each subject received two scores: anxious attachment style and avoidant attachment style. Higher scores indicated a more anxious or avoidant attachment style.

The Short UPPS-P Impulsive Behavior Scale was used to assess impulsivity (Billieux et al., 2012, Lynam, 2013). Only eight of this measure's 20 items were used to assess positive and negative urgency (4-point response scale), the two characteristics most often connected with addictive disorders. Each participant received two scores. It must be noted that the scale items were reverse-coded prior to the calculation of the two scores. High scores indicated more impulsivity.

In addition, intimate relationship satisfaction level was measured over the past year (9-point scale), higher scores reflecting greater satisfaction. The Short Happiness and Depression Scale (SDHS) (Joseph et al., 2004) measured participants' mood by using six 4-point items. One item with a 5-point response scale indicated loneliness (Rönkä et al., 2014), with greater loneliness indicated by higher scores. Self-esteem was measured on a 5-point Single-Item Self-Esteem Scale (SISE) (Robins et al., 2001). High scores reflect higher self-esteem.

Participants were also asked about childhood emotional or physical abuse. “When I was growing up, I believe that I was emotionally abused” was one of two questions for each form of abuse. Each abuse score was calculated from a 5-point scale ranging from “Never true” to “Very often true”.

##### 2.2.2.5 Violent and coercive sexuality (attitudes and experiences)

The short form of the Acceptance of Modern Myths about Sexual Aggression (AMMSA) (Helmke et al., 2014) measured rape myths and sexual aggression acceptance and is drawn from the 30-item tool of Gerger et al. (2007). This 11-item (7-point scale) measures participants' tolerance for rape myths and female sexual violence (e.g., “When a woman starts a relationship with a man, she must be aware that the man will assert his right to have sex”; “Many women tend to exaggerate the problem of male violence”). Overall scores were calculated for each participant. Higher ratings indicate greater myth acceptance.

The Sexual Experience Survey (SES) reported sexual perpetration and victimization in children older than 14 years (Koss et al., 2007, Testa et al., 2004). The 11-item victimization form determines whether a person was victimized (e.g. by touching, kissing, or rape). The 11-item perpetration form determines whether a person committed the same unwanted sexual actions. For this study, we assessed each participant's total perpetration and victimization scores. Coercive relationships and rape were also explored for perpetrators and victims.

### 2.3 Data analysis

First, for RQ1, we performed descriptive statistics (range, M, SD, frequency) on all research variables. Second, to answer RQ2, we performed a bivariate correlation analysis between the predictor and outcome variables. We also performed an analysis of variance (ANOVA) to test the effect of the nominal (no-ordered) predictor variables on the outcome variable. For both correlation and the ANOVA analyses, the significance level was set at < 0.05. In addition, since the data included in the current study had relatively high number of variables (56) and that we wanted to rank-order their predictive importance, we chose to conduct machine learning (ML) multivariate regression analysis (using the Extreme Gradient Boosting algorithm [XGBoost, R package]), instead of traditional linear regression, to solve RQ2. ML models are essentially predictive (for detail on how ML works see (D'Agostino, 2022; Sarker, 2021). They are constructed in two phases: the learning stage where the model analyzes and “learn” from the variables associations/relations; and the second stage where the model uses the “learned knowledge” to predict (D'Agostino, 2022; Sarker, 2021). The rationale for using ML algorithms rather than standard statistical methods relies on the fact that ML algorithms have hyperparameters allowing us to build and test different models in terms of prediction capabilities and to choose the best prediction models according to specific metrics. Furthermore, in contrast with standard linear regression models, most ML algorithm (including the one we used) are nonparametric—they do not impose a particular structure on the data. As such, they can capture nonlinear relationships, including interactions among the predictors themselves. Finally, compared with traditional regression, the machine learning algorithm we used is considered robust for high-dimensional data scenarios (the current study includes a relatively important number of predictors [56]), due to its ensemble nature (separately bootstrapping thousands of decision trees, then averaging their results) (D'Agostino, 2022; Sarker, 2021). The use of ML to predict health behavior or health outcomes have been growing in recent years (Weissler et al., 2021). For instance, in past three years, researchers used ML to predict fibromyalgia diagnose (Vera Cruz et al., 2021), to predict the use of smartphone health applications (Aboujaoude et al., 2022; Vera Cruz et al., 2023), to predict smoking cessation/reduction (Etter et al., 2023; Vera Cruz et al., 2023), to predict subjective well-being (Vera Cruz et al., 2023), and to predict online dating apps problematic use (Vera Cruz et al., 2023). Regarding the specific ML algorithm used to conduct the current study data analysis (XGBoost, Chen & Guestrin, 2016), it is based on decision trees. Decision trees (Loh, 2014) are statistical algorithms that create predictions based on particular conditions (see Loh, 2015, for an extensive and easy-to-understand PowerPoint explanation). Thus, the XGBoost algorithm processes data by aggregating predictions from numerous decision trees by using majority voting. After building the initial model from a set of decision trees and calculating the residuals (errors) for each observation in the dataset, XGBoost generates a new model to anticipate those errors, learns from them, and builds a better model. XGBoost iteratively adds weight to instances with incorrect predictions, learns from prior mistakes, develops new models, and combines them into an ensemble model with improved prediction skills. XGBoost is an ensemble learning regression and classification tool. Many configurable hyperparameters in XGBoost can improve model fitting. It is robust and can handle multiple data types and complex distributions. Most data scientists use XGBoost, which has won multiple data analysis competitions (Chen and Guestrin, 2016, Morde and Setty, 2019). XGBoost models output the relevance of each predictor variable by using Gain. Gain is the relative contribution of each feature (in this case, each predictor variable) to the model, calculated by taking its contribution for each tree. Gain values run from 0 to 1, which can be thought of as a percentage. A greater value for this metric when compared with another feature indicates that it is more significant for creating a prediction. The present analysis included only 49 predictor variables. Indeed, after multicollinearity testing (using the Random Forest algorithm), 7 of 56 predictors were excluded. The list of excluded variables is presented in the Appendix, Table B. ML requires splitting the dataset into at least two sets: one to train the model (typically 70–80 % of the sample) and the other to assess the model's prediction performance (20–30 %). In the current study, we divided the dataset as follows: train-set = 70 %; test-set = 30 %. The XGBoost parameters that we grid-tuned are: nrounds = c(500,1000,1500); max_depth = c(2,4,6); eta = c(0.025,0.05,0.1,0.3); gamma = c(0, 0.05, 0.1, 0.5, 0.7, 0.9, 1.0); colsample_bytree = c(0.4, 0.6, 0.8, 1.0); min_child_weight = c(1,2,3); subsample = 1. The result of this analysis is shown in Table 3.

## 3 Results

### 3.1 Descriptive statistics

Table 1 shows descriptive statistics for all study variables. The participants' age distribution (SD = 10.84) is off center from the mean (M = 33.18), showing a diverse age range of 18–75 years. Male participants (63.1 %) outnumbered female participants (35.2 %), and nonbinary participants represented less than 2 % of the sample. Most participants were heterosexual (77.6 %) and in a relationship, whether married or not (67.4 %).

**Table 1:** Descriptive statistic related to all study variables (5 categories, 1 outcome, 56 predictors).

**Variable categories / variables**	**Scale/Range**	**Mean/Frequency**	**SD**
**Outcome variable**			
Compulsive Cyberporn Use (CCU) (total score)	1–5	2.44(Male = 2.58; Female = 2.18)	0.93
**Socio-demographic characteristics (4 variables)**			
Age	18–75	33.18	10.84
Gender	*	Male = 1000 (63.1 %)Female = 557 (35.2 %)Non-binary = 27 (1.7 %)	
Marital status	*	Single = 509 (32.1 %)In relation, not married = 677 (42.7 %)In relation, married = 390 (24.6 %)Widow(er) = 8 (0.5%)	
Sexual orientation	*	Heterosexual = 1229 (77.6 %)Homosexual = 100 (6.3 %)Bisexual = 217 (13.7 %)Other = 38 (2.4 %)	
**Cyberporn use patterns (27 variables)**			
Weekly cyberporn use duration (CUD)	1 h-40 h	2.43	3.05
Frequency of cyberporn use (FCU) over the past year	1–11	5.39	2.76
Evolution of the cyberporn use since the Covid-19 period^a^	1–7	4.27	1.40
Paying subscriptions for cyberporn use	*	No = 1525 (96.3 %)Yes = 59 (3.7 %)	
Paying specific items for cyberporn use	1–6	1.15	0.50
Pornography negative moral perception	1–7	2.23	1.60
Cyberporn use improved the participants’ romantic live	1–5	2.51	1.00
Cyberporn use improved the participants’ sexual live^a^	1–5	2.69	1.07
Aroused by “domination” pornographic scenes^a^	1–4	2.62	1.05
Aroused by “humiliation” pornographic scenes	1–4	1.72	1.01
Aroused by “submission” pornographic scenes^a^	1–4	2.61	1.03
Aroused by “romantic love” pornographic scenes^a^	1–4	2.91	0.94
Aroused by “soft” pornographic scenes^a^	1–4	2.67	0.94
Aroused by “groups with many males” pornographic scenes	1–4	1.97	1.10
Aroused by “groups with many females” porn scenes^a^	1–4	2.66	1.07
Aroused by “young people” pornographic scenes^a^	1–4	2.72	0.99
Aroused by “old people” pornographic scenes	1–4	1.73	0.92
Aroused by “stories and dialogues” pornographic scenes^a^	1–4	2.41	1.01
Pornographic craving experience assessed with the PCE-S (total score)	0–10	4.48	2.21
PUMS sexual pleasure^a^	1–7	5.18	1.31
PUMS sexual curiosity	1–7	2.80	1.51
PUMS fantasy	1–7	3.44	1.64
PUMS boredom avoidance^a^	1–7	3.68	1.61
PUMS lack of sexual satisfaction	1–7	3.40	1.70
PUMS emotional distraction or suppression	1–7	2.91	1.64
PUMS stress reduction^a^	1–7	3.78	1.66
PUMS self-exploration	1–7	3.51	1.63
**Sexual dimensions (8 variables)**			
SFS dominance sexual motive^a^	1–4	2.78	0.66
SFS submission sexual motive^a^	1–4	2.58	0.62
SDI dyadic sexual desire^a^	0–8	5.03	1.65
SDI solitary sexual desire^a^	0–8	4.31	1.81
Number of sexual partners in the last 30 days	0–20	0.68	0.90
Number of sexual intercourses in the last 30 days	0–50	4.15	6.73
Sexual self-esteem^a^	1–4	2.33	0.90
Sexual satisfaction over the past year^a^	1–9	5.00	2.34
**Psychosocial and psychological dimensions (10 variables)**			
ECR-S anxious attachment style^a^	1–7	3.73	1.26
ECR-S avoidant attachment style^a^	1–7	3.60	0.84
UPPS-P negative urgency impulsivity^a^	1–4	2.54	0.66
UPPS-P positive urgency impulsivity^a^	1–4	2.50	0.59
Intimate relationship satisfaction over the past year^a^	1–9	5.28	2.80
SDHS depressive mood	1–4	1.23	0.75
Loneliness^a^	1–5	2.96	1.43
SISE self-esteem^a^	1–4	2.34	0.91
Childhood emotional abuse	0–4	1.22	1.38
Childhood physical abuse	0–4	0.67	1.08
**Violent and coercive sexuality (attitudes and experiences) (7 variables)**			
AMMSA acceptance of sexual aggression myths (total score)	1–7	2.76	1.34
SES-P perpetration (total score)	0–77	1.32	5.77
SES-P coercive relationships	*	No = 1445 (91.2 %)Yes = 139 (8.8 %)[male = 104 (74.83 %)]	
SES-P rape	*	No = 1444 (91.2 %)Yes = 140 (8.9 %)[male = 102 (72.86 %)]	
SES-V victimization (total score)	0–105	4.18	11.02
SES-V coercive relationships	*	No = 1286 (81.2 %)Yes = 298 (18.8 %)[female = 215 (72.14 %)]	
SES-V rape	*	No = 1214 (76.6 %)Yes = 370 (23.3 %)[female = 240 (64.86 %)]	

*Notes.* N = Number of participants, SD = Standard deviation.

*non-ordered categorical variable.

SDI = Sexual Desire Inventory; SFS = Sexual Function Scale; PUMS = Pornography Use Motivations Scale; SISE = Single-Item Self-Esteem Scale; AMMSA = Acceptance of Modern Myths about Sexual Aggression; SES-P = Sexual Experience Survey – Perpetration; SES-V = Sexual Experience Survey – Victimization; UPPS-P = Urgency, Premeditation, Perseverance, Sensation Seeking, Positive Urgency Impulsive Behavior Scale; ECR-S = Experience in Close Relationships – Short form; SDHS = Short Depression-Happiness Scale.

For the 25 predictor variables with “a” exponent (see Table 1), the mean scores were above the middle of the scale on their respective measures.

Among the participants, 3.7 % paid a subscription to watch cyberporn. Most sexual coercion (n = 104) and rape (n = 102) perpetrators were male (74.83 % and 72.86 %, respectively). Participants who reported sexual coercion (n = 215) and rape (n = 240) were mostly women (72.14 % and 64.86 %, respectively).

The mean score for the CCU outcome variable (2.44, SD = 0.93, median = 2.37) was close to the middle (2.5) of its 5-point scale. Percentiles were as follows: 25th = 1.63, 50th = 2.38, 75th = 3.13. Participant distribution on the first quartile score (CCU ≤ 1.63; n = 403) by sex was male = 168(41.68 %), female = 224(55.58 %), and nonbinary = 11(2.72 %); on the fourth quartile score (CCU > 3.13; n = 348) the distribution by sex was male = 246(70.68 %), female = 96(27.58 %), and nonbinary = 6(1.72 %).

The overall descriptive and inference results by sex are presented in Table 2.

**Table 2:** The study continuo and ordinal variables: Descriptive and inference statistics by sex.

**Variable categories / variables**	**Scale/Range**	**Mean male**	**SD** **male**	**Mean female**	**SD female**	***t***	***p*-value**
**Outcome variable**							
Compulsive Cyberporn Use (CCU) (total score)	1–5	2.58	0.88	2.18	0.97	8.05	0.003**
**Socio-demographic characteristics (4 variables)**							
Age	18–75	34.67	11.456	30.87	9.26	7.11	<0.001**
**Cyberporn use patterns (27 variables)**							
Weekly cyberporn use duration (CUD)	1 h-40 h	2.90	3.31	1.58	2.32	9.23	<0.001**
Frequency of cyberporn use (FCU) over the past year	1–11	6.06	2.82	4.16	2.16	14.81	<0.001**
Evolution of the cyberporn use since the Covid-19 period	1–7	4.43	1.39	4.01	1.37	5.70	0.001**
Paying specific items for cyberporn use	1–6	1.18	0.54	1.11	0.43	2.55	<0.001**
Pornography negative moral perception	1–7	2.21	1.62	2.30	1.57	−1.14	0.748
Cyberporn use improved the participants’ romantic live	1–5	2.45	0.99	2.63	1.00	−3.27	0.058
Cyberporn use improved the participants’ sexual live	1–5	2.61	1.07	2.84	1.07	−4.12	0.007**
Aroused by “domination” pornographic style	1–4	2.48	1.05	2.85	1.02	−6.59	<0.001**
Aroused by “humiliation” pornographic style	1–4	1.74	1.01	1.66	1.02	1.39	0.828
Aroused by “submission” pornographic style	1–4	2.51	1.05	2.77	0.98	−5.00	<0.001**
Aroused by “romantic love” pornographic style	1–4	2.89	0.92	2.94	0.99	−0.890	0.024*
Aroused by “soft” pornographic style	1–4	2.62	0.91	2.75	0.99	−2.40	0.024*
Aroused by “groups with many males” pornographic style	1–4	1.83	1.05	2.18	1.13	−5.93	<0.001**
Aroused by “groups with many females” porn style	1–4	2.83	1.04	2.34	1.08	8.71	0.002**
Aroused by “young people” pornographic style	1–4	2.88	0.93	2.41	1.03	8.96	<0.001**
Aroused by “old people” pornographic style	1–4	1.80	0.94	1.60	0.88	4.23	0.021*
Aroused by “stories and dialogues” pornographic style	1–4	2.41	1.01	2.39	1.01	0.36	0.921
Pornographic craving experience assessed with the PCE-S (total score)	0–10	4.72	2.05	4.08	2.41	5.24	<0.001**
PUMS sexual pleasure	1–7	5.34	1.22	4.89	1.43	6.21	<0.001**
PUMS sexual curiosity	1–7	2.79	1.47	2.86	1.58	−0.88	0.004**
PUMS fantasy	1–7	3.66	1.61	3.05	1.64	7.06	0.139
PUMS boredom avoidance	1–7	3.93	1.56	3.21	1.60	8.634	0.227
PUMS lack of sexual satisfaction	1–7	3.65	1.70	2.97	1.61	7.89	0.203
PUMS emotional distraction or suppression	1–7	3.16	1.67	2.46	1.49	8.54	0.002**
PUMS stress reduction	1–7	4.02	1.61	3.36	1.67	7.45	0.043*
PUMS self-exploration	1–7	3.46	1.61	3.61	1.68	−1.742	0.120
**Sexual dimensions (8 variables)**							
SFS domination sexual motive	1–4	2.73	0.67	2.85	0.65	−3.63	0.161
SFS submission sexual motive	1–4	2.66	0.60	2.44	0.62	6.89	0.739
SDI dyadic sexual desire	0–8	5.30	1.52	4.60	1.74	7.90	<0.001**
SDI solitary sexual desire	0–8	4.49	1.70	3.94	1.95	5.49	<0.001**
Number of sexual partners in the last 30 days	0–20	0.67	0.96	0.70	0.75	−0.55	0.077
Number of sexual intercourses in the last 30 days	0–50	3.88	6.17	4.72	7.67	−2.19	0.002**
Sexual self-esteem	1–4	2.35	0.87	2.30	0.94	1.09	0.016*
Sexual satisfaction over the past year	1–9	4.85	2.34	5.31	2.31	−3.77	0.379
**Psychosocial and psychological dimensions (10 variables)**							
ECR-S anxious attachment style	1–7	3.58	1.23	3.97	1.25	−5.91	0.588
ECR-S avoidant attachment style	1–7	3.54	0.81	3.68	0.87	−3.23	0.010*
UPPS-P negative urgency impulsivity	1–4	2.63	0.65	2.40	0.65	6.61	0.886
UPPS-P positive urgency impulsivity	1–4	2.55	0.58	2.42	0.60	4.33	0.180
Intimate relationship satisfaction over the past year	1–9	5.12	2.77	5.55	2.82	−2.86	0.488
SDHS depressive mood	1–4	1.22	0.76	1.23	0.71	−0.40	0.074
Loneliness	1–5	2.96	1.42	2.94	1.45	0.25	0.069
SISE self-esteem	1–4	2.41	0.89	2.25	0.92	3.35	0.593
Childhood emotional abuse	0–4	0.93	1.25	1.65	1.44	−9.79	<0.001**
Childhood physical abuse	0–4	0.49	0.92	0.96	1.28	−7.54	<0.001**
**Violent and coercive sexuality (attitudes and experiences) (7 variables)**							
AMMSA acceptance of sexual aggression myths (total score)	1–7	3.08	1.32	2.25	1.20	12.46	<0.001**
SES-P perpetration (total score)	0–77	1.63	6.68	0.81	3.78	3.10	<0.001**
SES-V victimization (total score)	0–105	1.37	4.50	9.09	16.19	−11.00	<0.001**

*Notes*. Male participants number = 1000; Female participants number = 557. SD = standard deviation; *t* = *t*-test statistics; * = significant at < 0.05; ** = significant at < 0.01. SDI = Sexual Desire Inventory; SFS = Sexual Function Scale; PUMS = Pornography Use Motivations Scale; SISE = Single-Item Self-Esteem Scale; AMMSA = Acceptance of Modern Myths about Sexual Aggression; SES-P = Sexual Experience Survey – Perpetration; SES-V = Sexual Experience Survey – Victimization; UPPS-P = Urgency, Premeditation, Perseverance, Sensation Seeking, Positive Urgency Impulsive Behavior Scale; ECR-S = Experience in Close Relationships – Short form; SDHS = Short Depression-Happiness Scale.

### 3.2 Correlation and ANOVA statistics

Table 3 shows the bivariate correlation statistics between all predictor factors and outcome. To interpret the correlations coefficients (r) values, a threshold widely used in behavioral sciences is the one proposed by Cohen (1988): r < 0.1, very small; 0.1 <= r < 0.3, small; 0.3 <= r < 0.5, moderate; r >= 0.5, large. Thus, based on these indices, Table 2 shows that most predictor variables are not strongly associated with the outcome variable. Eleven predictor variables had a moderate association with the outcome. These variables were the strength of pornography craving experiences (r = 0.50), suppression of negative emotions porn use motive (r = 0.49), stress reduction porn use motive (r = 0.42), frequency of cyberporn use (FCU) over the past year (r = 0.42), boredom avoidance porn use motive (r = 0.41), fantasy porn use motive (r = 0.39), lack of sexual satisfaction porn use motive (r = 0.37), self-exploration porn use motive (r = 0.34), dominance sexual motive (r = 0.33), sexual pleasure porn use motive (r = 0.31), and acceptance of rape myths and sexual aggression (r = 0.30). Nine predictor variables had small (r = 0.20 - 0.30) association with the outcome (see Table 2). All of these indicators were positively associated with the outcome; thus, as their values rose, so did the CCU scores.

**Table 3:** Bivariate correlations between the 49 independent variables and the participants’ CCU scores.

**Variable categories / variables**	***r***	**95 % CI**	
		Lower CI	Upper CI
**Sociodemographic (4 variables)**			
Age	-0.15	-0.195	-0.099
Gender	*		
Marital status	*		
Sexual orientation	*		
**Cyberporn use patterns (27 variables)**			
Weekly cyberporn use duration (CUD)	0.29	0.243	0.333
Frequency of cyberporn use (FCU) over the past year	0.42	0.379	0.460
Evolution of the cyberporn use since the Covid-19 period	0.28	0.233	0.324
Paying specific items for cyberporn use	0.19	0.146	0.240
Pornography negative moral perception	0.11	0.056	0.153
Cyberporn use improved the participants’ romantic live	-0.01	-0.063	0.036
Aroused by “domination” pornographic scenes	0.07	0.024	0.122
Aroused by “humiliation” pornographic scenes	0.11	0.062	0.159
Aroused by “submission” pornographic scenes	0.13	0.078	0.175
Aroused by “romantic love” pornographic scenes	0.06	0.014	0.112
Aroused by “soft” pornographic scenes	0.07	0.022	0.120
Aroused by “groups with many males” pornographic scenes	0.06	0.011	0.110
Aroused by “groups with many females” porn scenes	0.09	0.044	0.142
Aroused by “young people” pornographic scenes	0.18	0.132	0.227
Aroused by “old people” pornographic scenes	0.11	0.061	0.159
Aroused by “stories and dialogues” pornographic scenes	0.08	0.035	0.133
Pornographic craving experience assessed with the PCE-S (total score)	0.50	0.463	0.537
PUMS sexual pleasure	0.31	0.269	0.358
PUMS sexual curiosity	0.257	0.211	0.303
PUMS fantasy	0.39	0.342	0.426
PUMS boredom avoidance	0.41	0.364	0.446
PUMS lack of sexual satisfaction	0.37	0.323	0.408
PUMS emotional distraction or suppression	0.49	0.449	0.524
PUMS self-exploration	0.34	0.297	0.385
**Sexual dimensions (8 variables)**			
SFS dominance sexual motive	0.33	-0.375	-0.287
SFS submission sexual motive	0.25	-0.293	-0.201
SDI dyadic sexual desire	0.21	0.161	0.256
SDI solitary sexual desire	0.27	0.227	0.318
Number of sexual partners in the last 30 days	0.01	-0.036	0.063
Number of sexual intercourses in the last 30 days	-0.01	-0.055	0.043
Sexual self-esteem	-0.04	-0.090	0.008
Sexual satisfaction over the past year	-0.05	-0.102	-0.003
**Psychosocial and psychological dimensions (10 variables)**			
ECR-S anxious attachment style	0.26	0.210	0.302
ECR-S avoidant attachment style	0.20	0.151	0.246
UPPS-P negative urgency impulsivity	0.18	-0.230	-0.134
UPPS-P positive urgency impulsivity	0.22	-0.261	-0.167
Intimate relationship satisfaction over the past year	-0.12	-0.163	-0.066
SDHS depressive mood	0.16	0.112	0.208
Loneliness	0.19	0.143	0.238
SISE self-esteem	-0.09	-0.143	-0.045
Childhood emotional abuse	0.02	-0.027	0.072
Childhood physical abuse	0.06	0.011	0.109
**Violent and coercive sexuality (attitudes and experiences) (7 variables)**			
AMMSA acceptance of sexual aggression myths (total score)	0.30	0.255	0.344
SES-P perpetration (total score)	0.11	0.059	0.156
SES-V victimization (total score)	0.00	-0.050	0.049

*Notes.* N = number of participants, *r* = correlation coefficient; CI = confidence interval. Significance level = <0.05.

To interpret *r* values, it must consider that: r < 0.1, very small; 0.1 <= r < 0.3, small; 0.3 <= r < 0.5, moderate; r >= 0.5, large.

*Non-ordered categorical independent variable. For these variables we conducted ANOVA (analysis of variance). Results of the ANOVA a presented in Result section.

CCU = Compulsive Cyberporn Use; SDI = Sexual Desire Inventory; SFS = Sexual Function Scale; PUMS = Pornography Use Motivations Scale; SISE = Single-Item Self-Esteem Scale; AMMSA = Acceptance of Modern Myths about Sexual Aggression; SES-P = Sexual Experience Survey – Perpetration; SES-V = Sexual Experience Survey – Victimization; UPPS-P = Urgency, Premeditation, Perseverance, Sensation Seeking, Positive Urgency Impulsive Behavior Scale; ECR-S = Experience in Close Relationships – Short form; SDHS = Short Depression-Happiness Scale.

The non-ordered categorical predictor variables were analyzed by using ANOVA. ANOVA results indicated that single participants (M = 2.52; SD = 0.96) had considerably greater CCU scores than did those in relationships (M = 2.39; SD = 0.92), F(1, 1574) = 6.31, p =.012, η^2^p =.004. Male participants (M = 2.58; SD = 0.87) had significantly higher CCU scores than did female participants (M = 2.18; SD = 0.97), F(1, 1559) = 69.74, p <.001, η^2^p =.043. The mean scores of heterosexual, homosexual, and bisexual participants did not differ statistically (2.44, SD = 0.93; 2.53, SD = 0.88; 2.39, SD = 0.96, respectively), F(1, 1580) = 0.80, p =.490, η^2^p =.002.

### 3.3 Machine learning multivariate regression results

Table 4 shows the ranking of the most relevant predictor variables of participants' CCU scores with a machine learning multivariate regression model. The train-set model performed as follows: percentage of the outcome explained by the predictors (R2) = 81.5 %; mean squared error (MSE) = 0.33. The test-set model performance was as follows: R2 = 74.6 %; MSE = 0.69.

**Table 4:** Predictors of CCU, ranked in decreasing order of importance (XGBoost machine learning regression model).

**Rank**	**Features (predictor variables)**	Gain*
1	Pornographic craving experience assessed with the PCE-S (total score)	0.272
2	PUMS emotional distraction or suppression	0.146
3	Frequency of cyberporn use (FCU) over the past year	0.057
4	AMMSA acceptance of sexual aggression myths (total score)	0.050
5	ECR-S anxious attachment style	0.039
6	PUMS boredom avoidance	0.038
7	Age	0.026
8	PUMS sexual pleasure	0.025
9	SFS submission sexual motive	0.025
10	Evolution of the cyberporn use since the Covid-19 period	0.025
11	SDI dyadic sexual desire	0.024
12	PUMS self-exploration	0.019
13	ECR-S avoidant attachment style	0.016
14	SDHS depressive mood	0.014
15	SDI solitary sexual desire	0.014
16	PUMS curiosity	0.013
17	PUMS fantasy	0.013
18	SES-V victimization (total score)	0.012
19	SFS dominance sexual motive	0.011
20	UPPS-P positive urgency impulsivity	0.010
21	Aroused by “domination” pornographic scenes	0.010
22	Aroused by “soft” pornographic scenes	0.009
23	PUMS lack of sexual satisfaction	0.009
24	Pornography negative moral perception	0.008
25	UPPS-P negative urgency impulsivity	0.008
26	Aroused by “young people” pornographic scenes	0.007
27	Intimate relationship satisfaction over the past year	0.007
28	Weekly cyberporn use duration (CUD)	0.006
29	Childhood emotional abuse	0.006
30	Sexual self-esteem	0.006
31	Loneliness	0.006
32	Number of sexual intercourses in the last 30 days	0.005
33	SES-P perpetration (total score)	0.005
34	Aroused by “groups with many males” pornographic scenes	0.004
35	Aroused by “submission” pornographic scenes	0.004
36	Aroused by “old people” pornographic scenes	0.004
37	Number of sexual partners in the last 30 days	0.003
38	Aroused by “groups with many females” porn scenes	0.003
39	Sexual satisfaction over the past year	0.003
40	SISE self-esteem	0.002
41	Aroused by “stories and dialogues” pornographic scenes	0.002
42	Aroused by “humiliation” pornographic scenes	0.002
43	Childhood physical abuse	0.002
44	Cyberporn use improved the participants’ romantic live	0.002
45	Gender	0.002
46	Paying specific items for cyberporn use	0.002
47	Marital status	0.001
48	Sexual orientation	0.000
49	Aroused by “romantic love” pornographic scenes	0.000

*Notes.* *Gain values vary from 0 to 1. For instance, the Gain = 0.272 that the associated predictor (Pornography craving experience) has 27 % contribution to the variance of outcome variable (CCU).

CCU = Compulsive Cyberporn Use; SDI = Sexual Desire Inventory; SFS = Sexual Function Scale; PUMS = Pornography Use Motivations Scale; SISE = Single-Item Self-Esteem Scale; AMMSA = Acceptance of Modern Myths about Sexual Aggression; SES-P = Sexual Experience Survey – Perpetration; SES-V = Sexual Experience Survey – Victimization; UPPS-P = Urgency, Premeditation, Perseverance, Sensation Seeking, Positive Urgency Impulsive Behavior Scale; ECR-S = Experience in Close Relationships – Short form; SDHS = Short Depression-Happiness Scale.

The most important predictor was the strength of pornography craving experiences. The less important predictor was the arousal degree of “romantic love” pornographic scenes. Among the 49 predictors included, the 20 most important in decreasing order were as follows: strength of pornography craving experiences, suppression of negative emotions porn use motive, FCU over the past year, acceptance of rape myths and sexual aggression, anxious attachment style, boredom avoidance porn use motive, age, sexual pleasure porn use motive, submission sexual motive, evolution of cyberporn use since the COVID-19 pandemic started, dyadic sexual desire, self-exploration porn use motive, avoidant attachment style, depressive mood, solitary sexual desire, curiosity porn use motive, fantasy porn use motive, sexual victimization experiences, dominance sexual motive, and positive urgence impulsivity. Age was negatively correlated with the outcome, whereas all other factors were positively correlated.

## 4 Discussion

In this study, we assessed CCU in a wide cohort of people who use cyberporn and determined the most essential CCU score predictors from a wide variety of characteristics.

### 4.1 Descriptive results

For remainder, all participants declared to be cyberporn users. In this regard, it is noteworthy that the percentage of women on the study sample is 35.2 %, which is close to the 36 % of women who visited Pornhub last year (Pornhub, 2022). The number of male participants (63.1 %) was twice that of female participants, confirming that male cyberporn users are overrepresented (Camilleri et al., 2021, Kumar et al., 2021, LeBlanc and Trottier, 2022, Studer et al., 2019).

On a 5-point scale, 21.96 % of subjects reported CCU scores ≥ 3.13 (fourth quartile), suggesting a tendency toward compulsive use. This figure exceeds most recent research findings (Ballester-Arnal et al., 2017, Camilleri et al., 2021, Kumar et al., 2021, LeBlanc and Trottier, 2022, Mennig et al., 2020). The variances may be partially due to the range of approaches. In some studies (e.g. Ballester-Arnal et al., 2017), the authors examined all Internet-based sexual activities, materials, and behaviors without focusing on cyberporn. Camilleri et al. (2021) used the same metric as we did to examine cyberporn use among students at an American university. In our study, we recruited a sample of people who recently used porn and provided statistics for a more general and diversified group from various cultures and countries.

### 4.2 The most important predictors of CCU scores

The most important predictors of the participants’ CCU scores can be synthetically regrouped into the following six categories.

*Craving and frequency of cyberporn use*. This category is predicted by the strength of pornographic cravings and the past-year Frequency of Cyberporn Use (FCU). CCU scores are higher in participants with stronger pornographic cravings and more frequent use. This is not surprising, as these characteristics are linked to compulsive porn viewing (Bőthe et al., 2019, Weinstein et al., 2015). To our knowledge, our study is the first to use the elaborated intrusion theory to measure pornography craving, revealing a more specific relationship between CCU and craving. This is in coherence with a recent revision of the Interaction of Person-Affect-Cognition-Execution (I-PACE) model, incorporating desire thinking theory and craving experience as cognitive processes contributing to CCU (Brandtner et al., 2021). Indeed, this updated model aims to explain internet-use disorders such as porn use disorder. Pornography cravings and the FCU may indicate a loss of control and increasing priority as basic components of compulsive behaviors. Prospective research may be necessary to examine craving and CCU scores.

*Negative emotions, feelings, and experiences*. This category has five predictors: suppression of negative emotions and boredom avoidance porn use motives, anxious attachment style, avoidant attachment style, depressive mood, and sexual victimization experiences. CCU scores were linked to these negative emotions, feelings, and experiences in this study, suggesting that the use of cyberporn during vulnerable times is linked to compulsive use. In addition to the FCU, the purpose for this use appears to be important to compulsive consumption, especially when this motive reflects negative feelings. Participants who consume pornography as a coping strategy seem to be more likely to use it compulsively. Previous research has presented cybersex as a coping mechanism (Ben Brahim et al., 2019). These findings are consistent with studies that have linked coping motives with addictive behaviors (Melodia et al., 2022; Rochat et al., 2024; Zanetta Dauriat et al., 2011). After some personal experiences, subjective reward expectations may vary across individuals and contexts, from gratification for porn use to rather negative reinforcement processes as suggested by the coping and escape motives life (Brand et al., 2019, Laier et al., 2018) in coherence with the I-PACE model; Brand et al. (2019). In addition, sexual “addiction” is associated with greater rates of mental health issues (Cleveland Clinic, 2022). Camilleri et al. (2021) and Levin et al. (2012) also linked problematic pornography consumption with mental health issues such as depression, anxiety, and stress. According to Varfi et al. (2019), addictive cybersex is a “function” of depression and avoidant attachment style.

In the present study, victims of violent sexual experiences seem to have a greater tendency toward CCU. Barrault et al. (2016) linked problematic cybersexual attitudes to traumatic events such as physical and sexual abuse before the age of 17. Negative physical and sexual experiences may raise the risk of negative feelings, which may increase the use of cyberporn as a coping method.

*Age*. This category is represented by one predictor: age. Results suggest that younger cyberporn users present higher CCU scores than older users do. This may be because younger people have more sexual desire and craving, perhaps partly because younger adults secrete more testosterone, the hormone that drives sexual desire (Van Anders, 2012).

*Violent, submissive, and dominant sexual attitudes*. Three predictors represent this category: acceptance of rape myths and sexual aggression, submission, and dominance sexual motives. Higher CCU scores were observed among respondents who sexual aggression against females and rape myths. Perhaps pornography viewers are drawn to content that depicts violence against female partners and reinforces male–female dominance-submission stereotypes. This may reinforce sexual aggression myths, likewise creating a compulsive need for pornographic scenes. The sexual scripts (Vera Cruz, 2020, Vera Cruz and Sheridan, 2022) that shape users' sexual behavior may be crucial. Submission and dominance sexual motives also seem to predict CCU scores in the present study. Some people may use cyberporn to fulfill their sexual dominance fantasies, and people who desire to be sexually submissive could use cyberporn to meet their needs. Sexually submissive or dominant participants are more likely to have high CCU scores. Future research may reveal that this link involves craving or tolerating “hard” porn scenes that depict acts ranging from submission and dominance to violence. Future research should look into potential moderators’ impacts related to sexual behaviors, trauma history, and comparable factors.

*Sexual pleasure and exploration*. This category has four predictors: sexual pleasure porn use motives, self-exploration, curiosity, and fantasy. Participants who score higher on these motives may consume more cyberporn. The reward system (Lembke, 2021) and the gratification role in addictive behaviors (Brand et al., 2019) may explain this relationship, in which meeting the initial “need” (pleasure, curiosity, fantasy, self-exploration) leads to “more need” and so on.

*COVID-19 effect*. Only one predictor represents this category: evolution of cyberporn consumption since the COVID-19 pandemic started. We found that this tenth-ranked predictor predicted CCU scores for study participants, with a rise in cyberporn consumption since the COVID crisis started meaning higher current CCU scores for participants. The COVID-19 period, especially during confinement, has been linked to boredom, stress, and anxiety (Xie et al., 2022). This may increase the need for pleasure in order to cope with psychological issues, and thus more craving for pornography consumption.

### 4.3 The effect of sociodemographic variables

In this study, single participants had considerably higher CCU scores than did those in relationships. Single participants may feel solitary because they lack sexual partners, which may increase their cyberporn use. An earlier study (Kumar et al., 2021) reported that undergraduate medical students, in any form of relationship, presented higher problematic porn use. These results may be specific to the population and these variations may be due to sample characteristics.

As in earlier studies (Camilleri et al., 2021, Kumar et al., 2021, LeBlanc and Trottier, 2022, Studer et al., 2019), male respondents had significantly higher CCU scores than did female participants. Participants' CCU scores were unaffected by sexual orientation.

### 4.4 The least important predictors of CCU scores

Some variables, such as impulsivity, predicted CCU scores less reliably. The literature disagrees on the role of this variable. According to Billieux et al. (2012), impulsivity helps induce and maintain addiction. Higher attentional impulsivity may lead to uncontrolled use of cyberporn (Antons et al., 2019). Impulsivity may not be as relevant, however, in problematic pornography consumption (Bőthe et al., 2019). Our study revealed how positive and negative urgency specifically affect cyberporn consumption. General versus domain-specific impulsivity may impact differently hypersexuality (Reid et al., 2015). Recent results failed however to indicate the impact of domain-specific impulsivity (e.g. sexual impulsivity) in hypersexuality (Carvalho et al., 2021). Further studies are still needed to asses such potential differences especially for compulsive cyberporn use, a domain influenced by specific stimuli-related reactivity (Love et al., 2015).

Sexual pleasure and sexual encounter numbers predicted CCU scores to a lesser extent. This would reduce the link between sexual satisfaction, encounter numbers, and CCU risk. Sexual activity and satisfaction seem to have little impact on the development of CCU.

A moderate association was found between pornography negative moral perception (moral incongruence) and CCU scores in this study. It is one of the least important CCU score predictors, ranking 24th out of 49. Lewczuk et al. (2020) noticed that moral incongruence is associated to compulsive porn consumption across religiosity. The present research did not assess religion and comprised a diverse sample from different countries and cultures. These considerations may help explain our nuanced results compared to earlier investigations (e.g. Grubbs et al., 2019, Lewczuk et al., 2020).

### 4.5 Limitations

One limitation of the study is that the recruitment approach does not reveal how much of the sample represents people with cyberporn use activities. Thus, generalizing the findings requires caution. Even though Prolific seems to provide Internet-representative samples (Antons et al., 2019), recruiting the sample on the basis of prior pornographic use may potentially limit interpretation and generalization. Although this recruitment criterion may aid in understanding recent pornographic use, it may not apply to everyone.

In addition, this cross-sectional exploratory investigation identified CCU score predictors from numerous factors. Longitudinal and hypothesis-testing research is needed to understand how psychological and psychosocial variables interfere with CCU.

Finally, the machine learning (ML) analysis could not be separately conducted for each sex. This can be considered as limitation. However, it is important to note that such analyses were note carried out because of the limitation regarding the number of participants. Indeed, to perform well, the machine learning algorithm we used need a consequent amount of data (D’Agostino, 2022; Sarker, 2021). If we had run two models separately (one for males = 1000 participants; one for females = 557 participants) each model would comprise a relatively “small” sample (as for machine learning standards); the male ML model would comprise almost twice the number of participants of the female model. Thus, comparing results from such imbalanced models would be problematic, given the machine learning “technicalities” (D’Agostino, 2022; Sarker, 2021).

## 5 Conclusions

Most previous research (Camilleri et al., 2021, Kumar et al., 2021, LeBlanc and Trottier, 2022, Studer et al., 2019) reported a lower percentage of participants with CCU scores than was found in this study. The five strongest predictors of CCU scores are strength of pornography experiences, suppression of negative emotions porn use motive, cyberporn use frequency over the last year, acceptance of rape myths, and anxious attachment style. This study adds to the CCU literature and may help clinicians treat and prevent CCU. Comparable survey design that targets different types of compulsive sexual behavior may help to enhance knowledge for other addictive behaviors.

Declarations.

Conflicts of Interest: The authors do not have any conflicts of interest to report.

Not applicable.

Ethics approval and consent to participate: Participants gave digital informed consent for their survey contribution. Participation was voluntary and restricted to those aged ≥ 18 years. All data were anonymously collected. The survey followed the [anonymized] Research Ethics Committee (2020–04-05).

Author contributions: Conceptualization: FBB, GVC, RC, YK. Methodology: FBB, GVC, RC, YK. Formal analysis: GVC. Original draft: FBB, GVC, YK. Review & editing: All authors.

Funding.

Availability of data and materials: The material used in this study and data supporting these findings can be obtained from the corresponding author upon request.

## CRediT authorship contribution statement

**Farah Ben Brahim:** Writing – review & editing, Writing – original draft, Methodology, Conceptualization. **Robert Courtois:** Writing – review & editing, Methodology, Conceptualization. **Germano Vera Cruz:** Writing – review & editing, Writing – original draft, Formal analysis. **Yasser Khazaal:** Writing – review & editing, Writing – original draft, Methodology, Conceptualization.

## Declaration of competing interest

The authors declare that they have no known competing financial interests or personal relationships that could have appeared to influence the work reported in this paper.

## Data Availability

Data will be made available on request.
